# Experimental Study on Ecological Performance Improvement of Sprayed Planting Concrete Based on the Addition of Polymer Composite Material

**DOI:** 10.3390/ijerph191912121

**Published:** 2022-09-25

**Authors:** Haoqiang Lai, Jiaxin Du, Cuiying Zhou, Zhen Liu

**Affiliations:** 1 School of Civil Engineering, Sun Yat-sen University, Guangzhou 510275, China; 2 Guangdong Engineering Research Center for Major Infrastructures Safety, Guangzhou 510275, China

**Keywords:** polymer composite material, sprayed planting concrete (SPC), ecological performance, improvement test, rocky steep slope

## Abstract

Sprayed planting concrete (SPC) can be used for the ecological restoration of rocky steep slopes. It is a kind of outside-soil material with excellent soil and slope stabilization performance, and plants can grow in SPC, thus achieving harmony between engineering stability and ecological restoration and improving the landscape and ecosystem. The addition of cement is the key to allowing SPC to achieve slope stabilization and prevent soil erosion. However, the addition of cement can cause SPC to have high alkalinity, overheating (cement generates hydration heat), and excessive hardening, which are not conducive to the growth of plants and can lead to poor ecological performance of SPC for slope ecological restoration. We studied the improvement of the ecological performance of SPC by using a polymer composite material composed of a polymer adhesive material and a polymer water-retaining material. This paper studied the improvement effects of the polymer composite material on the ecological performance of SPC used in slope ecological restoration through a laboratory erosion resistance test and a plant growth test. The results showed that SPC with the addition of polymer composite material can reduce its cement content by about 50% while still retaining excellent erosion resistance performance when it is used in slope ecological restoration. Additionally, the plant germination rates and plant heights when using the SPC improved by polymer composite material were increased by 190% and 110%, respectively. These results show that polymer composite material can significantly improve the ecological performance of SPC and effectively improve its slope ecological restoration effects. This study provides theoretical and technical support for the application of SPC in ecological restoration on rocky steep slopes.

## 1. Introduction

As many mining, expressway construction, and other engineering projects have been carried out, China’s economic development has been greatly promoted, but these projects have also led to great amounts of mountain excavation and have caused damage to the ecological environment of mountain slopes. Damaged mountain slopes are high and steep, and the slopes’ surfaces are rocky and free of plants. If damaged steep rocky slopes are not treated, there is a risk of latent dangers such as slope sliding, and this is not conducive to environmental protection. It is difficult to satisfy the ecological restoration requirements of rocky steep slopes with conventional outside-soil ecological restoration techniques. The use of sprayed planting concrete (SPC) in the ecological restoration of rocky steep slopes has been proven to be an effective and feasible scheme [1,2]. Due to the addition of cement, SPC has excellent soil and slope stabilization and erosion resistance performance, and has become a superb outside-soil material for ecological restoration of steep rocky slopes [3]. Cement can significantly improve the unconfined compressive strength and sheer strength of the soil [4,5]. According to a laboratory simulated rainfall scouring test, scholars have verified that the addition of cement can effectively enhance the scouring resistance of the slope and significantly reduce the soil loss of the slope [6]. However, the addition of cement can increase the alkalinity of the soil and generate a large amount of hydration heat [7], which is not conducive to plant growth [8,9]. Moreover, the excessive addition of cement can form an excessive hardening layer in the soil, which could inhibit the growth of plants and can also change the dry bulk density, compressive strength, and porosity of the soil. When too much cement is added, the porosity of the soil becomes too small and the soil’s condition cannot satisfy the requirements of plant growth [10,11]. The above studies show that SPC with the addition of cement has superb soil and slope stabilization and erosion resistance performance. Using SPC can restore the natural environment of the slope by using non-traditional engineering approaches, such as a lattice girder, and letting plants grow in SPC can achieve harmony between engineering stability and ecological restoration. This means that SPC can improve the landscape and the ecosystem while achieving mechanical safety and stability. However, the ecological performance of SPC still needs to be improved.

To solve the problem of SPC being adverse to plant growth, different studies regarding improving the ecological performance of SPC have been carried out which mainly focus on the problems of high alkalinity, overheating (cement generates hydration heat), and excessive hardening [12,13,14,15]. Given that reducing the cement content in SPC could alleviate these problems, we searched for ecological materials that could replace cement without compromising the mechanical properties of SPC such as erosion resistance performance. Additionally, the porosity of SPC could be increased by adding materials to further alleviate the problem of excessive hardening. For example, in order to alleviate the problems of excessive hardening and overheating of SPC, which are key factors closely related to the good growth and development of plants, water-retaining agents, sawdust, palm, and other materials could be added during the preparation of SPC to improve the porosity, water retention, and permeability of SPC [16,17,18]. The amount of cement added is the key factor that leads to problems with SPC, and these problems can cause serious adverse effects on plant growth and the ecological performance of SPC. Therefore, to improve the ecological performance of SPC, measures must be taken to reduce the additional amount of cement to alleviate the problems of high alkalinity, overheating, and excessive hardening. The addition of silica fume, gypsum, fly ash, phosphogypsum, and other gel materials could reduce the amount of cement added to SPC without compromising the mechanical properties of SPC to ease the problems of high alkalinity, overheating, and excessive hardening [19,20,21]. In addition, the use of low alkalinity sulphoaluminate cement is also an effective means of achieving this. For example, researchers have used clay silt, low-alkalinity inorganic gel materials, polyvinyl acetate lotion binder, a small amount of water-retaining agents, and compound fertilizer to prepare SPC, which resulted in good ecological performance and was conducive to plant growth [22]. The above research shows that reducing the cement content of SPC, improving its water retention and porosity, and reducing its alkalinity can effectively improve the ecological performance of SPC. Furthermore, we have provided valuable references for the study of this article. In short, how to effectively improve the ecological performance of SPC and improve the ecological slope restoration effect of SPC is an important problem to be solved. In order to allow SPC to become an ecological restoration material for rocky steep slopes with good ecological performance, the key is to improve the ecological performance of SPC while maintaining excellent erosion resistance performance and preventing water and soil loss [23].

In view of the poor ecological performance of SPC, this paper studies SPC’s performance and focuses on improving SPC by using a polymer composite material composed of a polymer adhesive material and a water-retaining material in order to achieve this study’s objectives of improving the ecological performance of SPC and making it a good ecological restoration material for slopes [24]. Polymer adhesive material and water-retaining material are green and environmentally friendly ecological materials, which can promote the growth and development of plants [25,26,27]. The addition of polymer adhesive material can reduce the cement content in the SPC to reduce alkalinity and hydration heat, thereby alleviating the problems of high alkalinity, overheating, and excessive hardening to a certain extent. The polymer water-retaining material can increase the porosity and water-retaining performance of the SPC, saving it from excessive hardening and creating favorable conditions for plant growth. In this paper, the effects of polymer composite materials on the improvement of SPC were studied through a laboratory erosion resistance test and a plant growth test. With the improvement of polymer composite materials, the ecological performance of SPC could be significantly improved while maintaining excellent erosion resistance performance. In this way, we can expect that SPC will become a good ecological restoration material for rocky steep slopes.

## 2. Experimental Content and Method

### 2.1. Experimental Purpose and Methodology

(1) Experimental purpose

The erosion resistance test reflects the mechanical properties of the material, such as soil and slope stabilization performance, and the plant growth test reflects the ecological performance of the material. We performed a plant experiment regarding the ratio of the polymer composite material to cement, and the improvement of the use of polymer composite material on the ecological performance of SPC was studied. Combined with a simulated rainfall erosion test, the influences of added polymer composite material on the erosion resistance performance of SPC were studied.

(2) Experimental methodology

**Figure 1:**
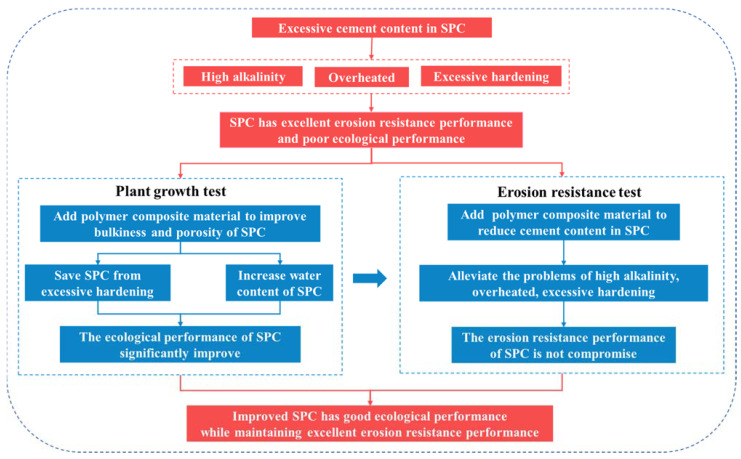
Experimental methodology.

This study focused on the use of polymer composite material to improve the ecological performance of SPC without compromising its erosion resistance performance. An excessive cement content could lead to high alkalinity and excessive hardening of the SPC, which could cause adverse effects on plant growth. The addition of polymer composite material can reduce the cement content of SPC and alleviate these problems, and can also improve its bulkiness, porosity, water retention, and storage capacity; in doing so, it can create good conditions for plant growth and development and can enhance the ecological performance of SPC. The improvement of the ecological performance of SPC was studied via a plant growth test. The polymer composite material with a good soil consolidation effect was added to reduce the addition of cement to SPC. The improvement effects of the polymer composite material on the erosion resistance performance of SPC were studied via an erosion resistance test [27] to determine whether the SPC has improved erosion resistance performance. Combined with the erosion resistance test and the plant growth test, we studied whether SPC could become a good ecological restoration material for slopes after the improvement of the polymer composite material. The experimental methodology is shown in Figure 1.

### 2.2. Material

The polymer composite materials used in this paper included a polymer adhesive material and a polymer water-retaining material, and the optimal material ratio of SPC was determined and optimized according to the plant growth test and erosion resistance test.

The polymer adhesive material is a type of nano water-based organic adhesive with a molecular formula of [CH2CHCOOCH_3_], whose main component is polyvinyl acetate. It is a white viscous emulsion at normal temperature, resistant to hydrolysis, and insoluble in water, but it has good dispersibility in water. Polymer adhesive material is often used in dispersions prepared with water, and it has a high viscosity and soil consolidation. The material is also degradable, with a degradation cycle of 24 months (the long-term resistance will be researched next) under natural conditions, and will be degraded into CO_2_ and H_2_O without generating any hazardous substances [28,29,30].

Polymer water-retaining material has excellent water-absorption and water-retention performance, and its main component is sodium polyacrylate; the molecular formula is C_3_H_3_NaO_2_. The water-retaining material consists of fine white particles with a particle size of about 0.02mm under normal temperatures and dry conditions, and it becomes a form of transparent gel after absorbing water. When the material absorbs water to a saturated state its mass can reach up to 250 times that of its dry weight, its volume can increase 2500-fold, and it can release water and shrink to near its original volume [31]. Additionally, the water absorption–release and shrinkage–expansion cycles of the material can be repeated until the material is degraded. The degradation period of this water-retaining material is 48 months and the final degradation products are CO_2_ and H_2_O, which do not produce any hazardous substances.

**Figure 2:**
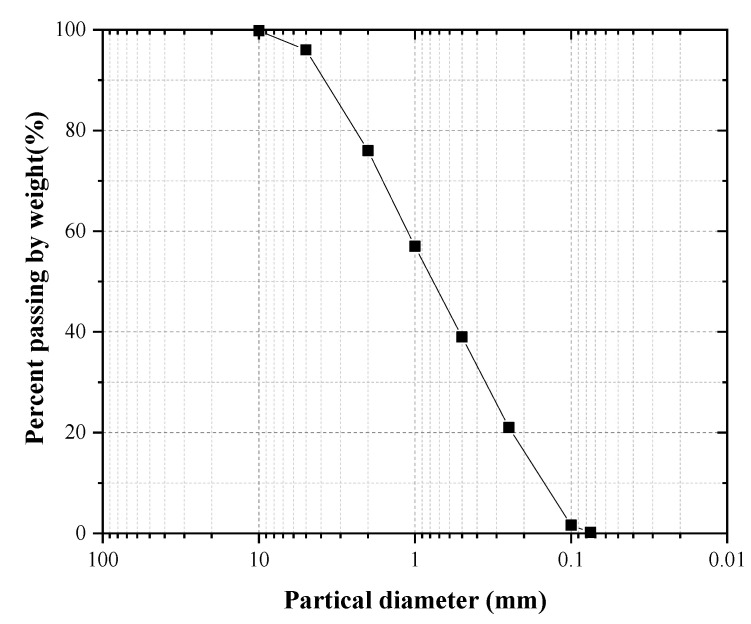
The particle grading curve.

SPC is made of ordinary HE portland cement and clayey silt from South China (the soil sample is typical red lateritic soil, which can be found in countries and regions on both sides of the Tropic of Cancer [32,33]. The particle grading curve of the tested soil sample is shown in Figure 2, and some of its physical properties are shown in Table 1). South China is a mountainous region where rocky steep slopes are widespread, and many of these urgently need to be ecologically restored due to excessive mountain excavation.

### 2.3. Content and Methods

#### 2.3.1. Plant Growth Test

Pigeon pea (Cajanus cajan), a plant that is frequently used for slope restoration in South China and which can grow in most tropical and subtropical regions of the world was used in the plant growth test as the test object. Each test group consisted of 400 g clayey silt, which was placed in a 15 cm-long, 12 cm-wide, and 6 cm-high plastic planting vessel. The materials were added to the soil samples of each group according to the designed material ratio. After fully and evenly mixing, 10 pigeon pea seeds were sown for each group, then we sprayed water into the soil and ensured that the water volume of each group was 50 mL. We placed the test groups in the laboratory where they were exposed to light and unified the maintenance conditions of all the test groups. We watered each sample with 50 mL of water once every other day for the first three days and once every three days thereafter to simulate dry conditions. We recorded the amount of germination, and germination of the plant seeds was recorded only when the germination exceeded 1 cm. We recorded the plant growth height once every two days, and the test period was 20 days in total. Three parallel tests were set for every test group, and we took the average value for the final test results. The plant germination rate was calculated as follows in Formula (1):

(1) $$\chi =\frac{a}{a_{0}}\times 100\%$$

where χ is the plant germination rate of the test group (%), a is the germination number of the test group, and a_0_ is the number of seeds sown in the test group.

(1) Plant growth test for the polymer composite material

The plant growth test for the polymer composite materials included 1 control group (CK) without any materials added and 19 test groups with different ratios of materials added. As shown in Table 2, the soil samples of test group A only contained the added polymer adhesive materials; the soil samples of test group B only contained the added polymer water-retaining materials; and the soil samples of group C contained the added polymer composite materials (polymer adhesive materials and polymer water-retaining materials) according to different ratios. Throughout this test, the effects of polymer composite materials on plant growth conditions of soil were explored.

(2) Plant growth test for improved SPC

There were 14 test groups with different added materials in the plant growth test for improved SPC, as shown in Table 3, including group D—the unimproved SPC, and group E—the improved SPC. By using these experiments, the improvement effects of polymer composite materials on the ecological performance of SPC were explored.

#### 2.3.2. Erosion Resistance Test

**Figure 3:**
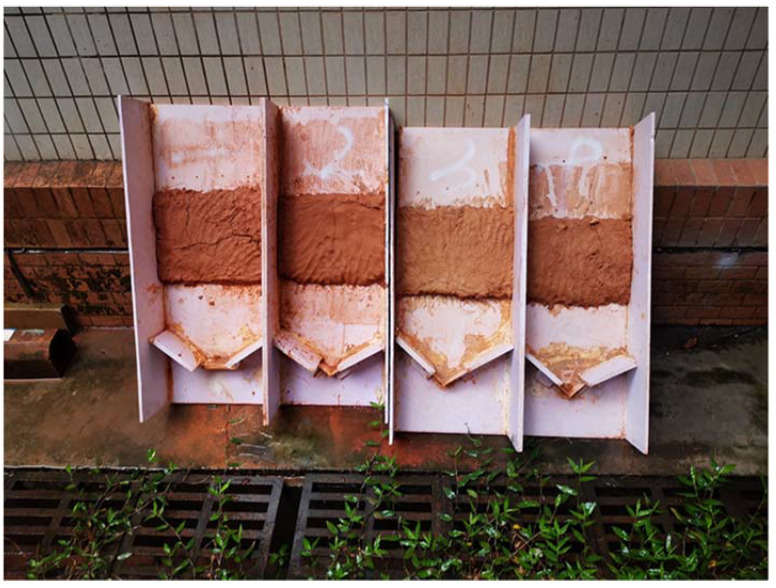
The simulated adjustable slope.

The erosion resistance test was carried out to study the effects of composite polymer material on the erosion resistance performance of SPC. The test equipment included a spray nozzle, a simulated slope, and a hot-air oven. We used the spray nozzle to spray tap water to simulate rainfall. The PVC plate was cut and bonded to make a soil bin with a length of 60 cm, a width of 35 cm, and a height of 30 cm to simulate the adjustable slope, as shown in Figure 3. The hot-air oven was used to dry the washed soil sample. We adjusted the simulated slope inclination to 70°, as shown in Table 4, to simulate a steep slope in the erosion resistance test. The variables of the test were the material additions of outside soil on the simulated slope. There were 18 test groups in the erosion resistance test, including a control group without any added materials and 17 test groups with different material additions, as shown in Table 2 and Table 3. The soil samples of test group A only contained the added polymer adhesive materials; group D represented the unimproved SPC, and group E represented the improved SPC.

The erosion resistance test was conducted in the laboratory. We took 3 kg of prepared soil samples for each group and prepared the polymer adhesive material dispersion with a concentration of 10%. We then mixed the polymer adhesive materials, cement, and polymer water-retaining materials into the soil sample according to the material ratios given in Table 2 and Table 3. We ensured that the soil-to-water ratio of each test group was 8:1. The prepared soil samples were applied to the simulated slope in three layers in the same way as that used for the outside soil spraying to a thickness of 9 cm. The simulated slope covered with the tested soil samples was maintained at room temperature for 72 h, and then the test was started. The slope was set to 70°. The rainfall intensity simulated in the test was 150 mm/h (this rainfall intensity is classified as torrential rain according to the standards of the National Meteorological Administration), which simulated the torrential rain that occurs in South China. After erosion, the remaining washed soil samples on the slope were collected and placed in the oven for drying. The remaining dried and washed soil samples were weighed.

The erosion rate was used to evaluate the erosion resistance performance of the tested soil samples on the slopes. The smaller the erosion rate, the stronger the erosion resistance performance of the tested soil samples on the slopes and the better the soil consolidation effect of the added materials. The erosion rate *E_a_* is the ratio of the dry weight of the scoured soil to the dry weight of the soil sample before the test. The erosion rate was calculated as follows in Formula (2):

(2) $$E_{a}=\frac{m-\Delta m}{m}\times 100\%$$

where *m* is the dry weight of the soil sample before the test (g), and Δ*m* is the dry weight of the scoured soil (g).

## 3. Results and Discussion

### 3.1. Plant Growth Test

#### 3.1.1. Results of the Plant Growth Test with Polymer Composite Material

**Figure 4:**
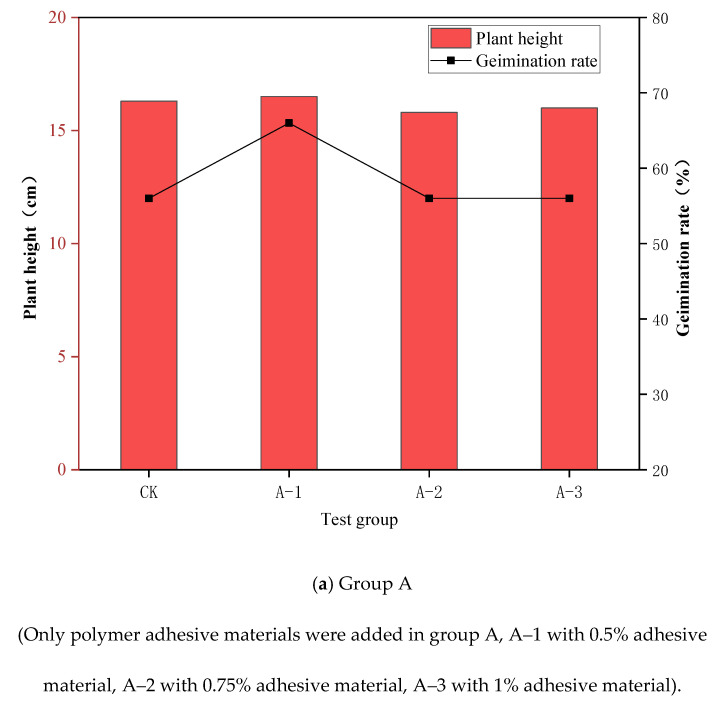

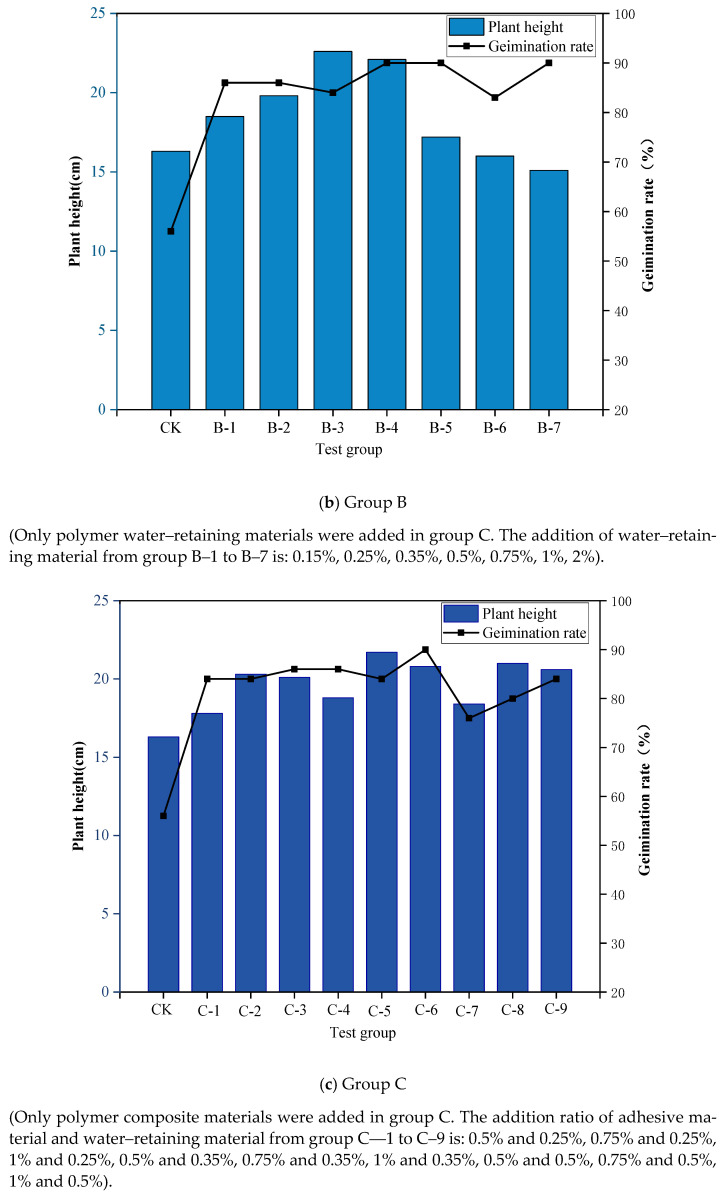
Result of the plant growth test with polymer composite material.

The combination of polymer adhesive material and polymer water-retaining material could promote plant growth. Figure 4 shows the plant germination rate and plant height of the control group CK, group A (only the polymer adhesive material was added), group B (only the polymer water-retaining material was added), and group C (the polymer composite material was added) according to the plant growth test with the polymer composite material. According to the test results of group A, shown in Figure 4a, and compared with the control group CK without the addition of any materials, the germination rate and plant height of the plants were not compromised with the addition of the polymer adhesive materials. It can be inferred that the addition of a certain amount of polymer adhesive materials in the soil does not impose adverse effects on plant growth. It can be seen from the test results of group B, shown in Figure 4b, that with the addition of polymer water-retaining materials the germination rate was significantly increased, and was 56% higher than that of control group CK; generally showing a rising trend with the increase in the amount of water-retaining material added. The germination rate of each test group with the water-retaining materials was up to approximately 90%. Comparing the plant heights at 20 days, the test results showed that a certain amount of polymer water-retaining material could effectively improve the height of plants. However, with the increase in the amount of polymer water-retaining material, the plant height first increased and then decreased. When the added polymer water-retaining material exceeded 1%, the materials began to display a negative effect on plant growth. In summary, polymer water-retaining material was found to effectively promote the growth of plants, but the amount added should be controlled within the appropriate range following specific conditions.

The polymer composite material was found to improve the plant growth conditions of the soil and improve its ecological performance. Figure 4c shows the germination rate and plant height at 20 days for group C with the addition of polymer composite material. It can be seen from the figure that, as compared with the plant growth of control group CK, the germination rate and plant height of test group C with the addition of the polymer adhesive material and water-retaining material were significantly higher than those of the control group. Additionally, the plant germination rate and plant height after the addition of the polymer composite material were higher than those in group B. It can be inferred that the polymer composite material can effectively promote plant growth.

#### 3.1.2. Results of the Plant Growth Test for Improved SPC

**Figure 5:**
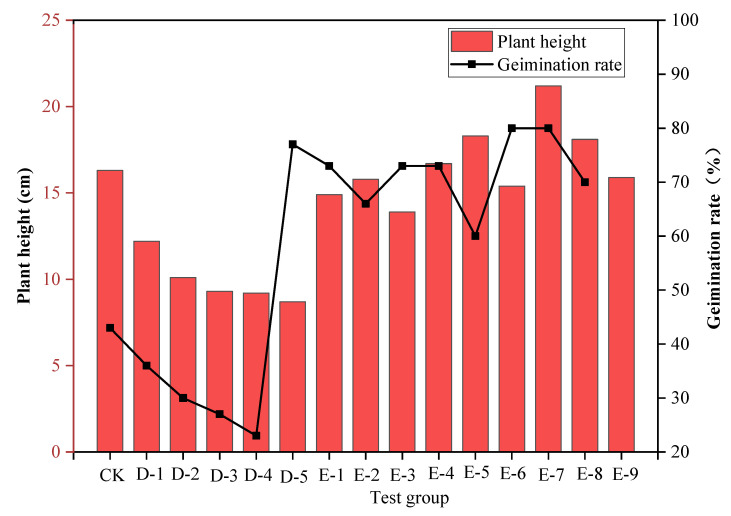
Result of plant growth test with improved SPC.

The addition of cement to the SPC inhibited plant growth. It can be seen from Figure 5 that the addition of cement was obviously unfavorable for plant growth. The plant germination rate of the unimproved SPC of group D was 23–43%, which was far lower than the plant germination rate (56%) and plant height (16.3 cm) of control group CK without the addition of any materials. It can be clearly seen that the addition of cement to the SPC inhibited plant germination and growth.

The polymer composite material was found to improve the plant growth conditions with the use of SPC and to improve the ecological performance of the SPC. To improve the plant growth conditions when using SPC and to improve its ecological performance, the polymer composite material was added to group E and the cement content in the improved SPC was markedly reduced. Comparing the germination rate and plant height when using the unimproved SPC in group D and the improved SPC in group E, as Figure 5 shows, the germination rate when using the improved SPC was increased by 200–300%, and the plant height was also significantly improved. Compared with control group CK, the germination rate when using the improved SPC in group E was generally higher than that of the control group CK. Compared with group B and group C with better ecological performance, the germination rate, and plant height when using the improved SPC in group E reached levels similar to those of group B and group C, among which the plant germination rates of group E-5 and E-6 reached 80%, and whose plant heights increased to 21.1 cm and 18.1 cm, respectively.

**Figure 6:**
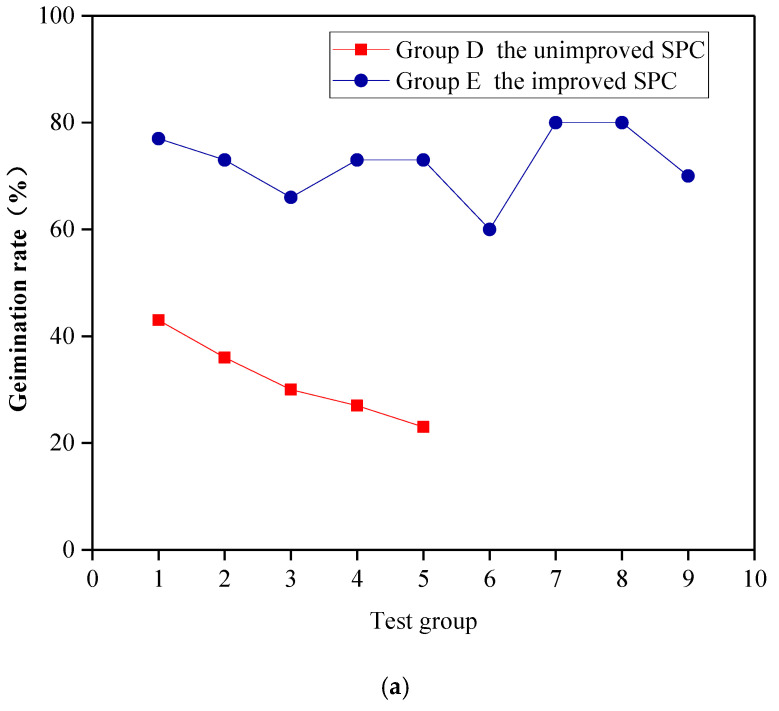

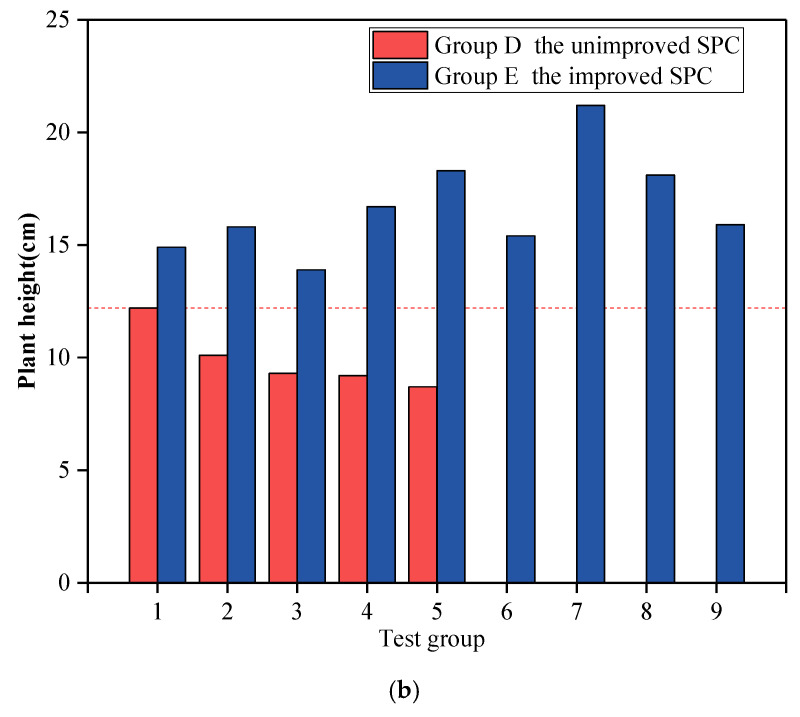
Plant growth with SPC before and after improvement. (**a**) Germination rates when using SPC before and after improvement. (**b**) Plant heights when using SPC before and after improvement.

According to the plant growth test with the improved SPC, the cement content of the improved SPC was reduced, the plant growth conditions were significantly improved, and the ecological performance was excellent. As Figure 6 shows, the germination rate when using the improved SPC increased by 200–300%, and the plant height was also significantly improved. The addition of the polymer adhesive material was found to reduce the cement content of SPC and alleviate the adverse effects of cement on plant growth. Moreover, the polymer water-retaining material has the characteristics of water swelling and water shrinkage, which can effectively improve the porosity and bulkiness of the SPC, alleviate the problem of excessive hardening, and promote seed germination and plant root growth. Furthermore, the addition of water-retaining material was found to improve the water-retaining capacity and water content of the SPC, ensuring that plants have sufficient water for growth and development, which could also alleviate the influence of hydration heat generated by cement on plant growth. In summary, the polymer adhesive material and polymer water-retaining material jointly created good plant growth conditions when using SPC. With the improvement of the polymer composite material, the ecological performance of the SPC was significantly improved; thus, SPC could become an excellent material for ecological slope restoration.

### 3.2. Erosion Resistance Test

#### 3.2.1. Results of the Erosion Resistance Test

**Figure 7:**
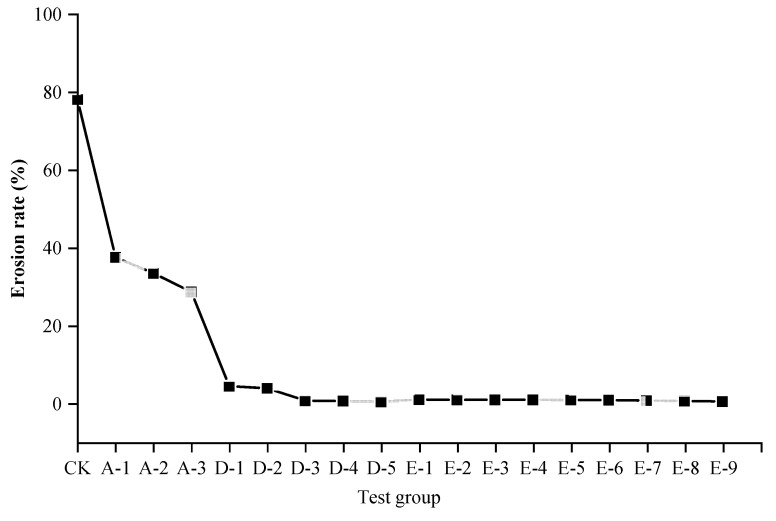
Results of the erosion resistance test.

SPC has excellent erosion resistance performance, and the addition of polymer composite material can reduce its cement content without compromising its erosion resistance performance. The results of the erosion resistance test are shown in Figure 7. The erosion rate of the control group CK without any material added was 78% at 15 min under simulated heavy rain conditions, and the erosion rates of the test groups A, D, and E were much lower than those of the control group CK, which leads to the conclusion that the addition of polymer adhesive material and cement can effectively improve the erosion resistance capacity of the slope’s outside soil, as the test results showed.

The erosion rate of the group A slope with only polymer adhesive material added was 28–37%. With an increase in the amount of polymer adhesive material, the erosion rate of the slope decreased. It is safe to infer that adding even a small amount of polymer adhesive material can effectively enhance the erosion resistance of the slope, and polymer adhesive material was found to be an excellent soil consolidation material. It can be seen from Figure 7 that the erosion rate of the unimproved SPC with different amounts of cement added in group D was only 0.3–4.3%. Compared with the control group CK, the erosion rate of the tested slope surface was reduced by 94.5–99.6%; by inference, cement had a significant soil consolidation effect. However, as mentioned above, the addition of cement can compromise the ecological performance of SPC. Additionally, as Figure 7 demonstrates, the erosion rate of the improved SPC in group E after the amelioration of the polymer composite material was 0.38–0.85%, which was similar to or even lower than that of the unimproved SPC in group D. Thus, the conclusion can be drawn that the cement content of the SPC can be reduced by 50% after the improvement of the polymer composite material, while still achieving the same or even better erosion resistance performance

**Figure 8:**
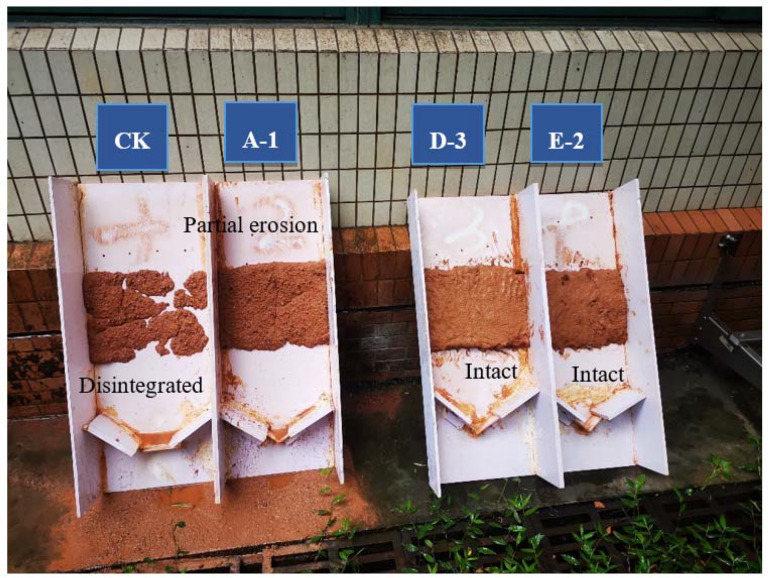
Condition of the typical simulated slope after 15 min erosion.

Several typical tested slopes after 15 min of simulated rainfall scouring are presented in Figure 8. It can be seen that the soil samples of the control group CK without any materials added on the simulated steep rocky slope had been scoured and washed away until they disintegrated, and the soil samples of group A-1 with only polymer adhesive material added were merely scoured to partial erosion. The tested slope of group A-1 was stable overall, which could lead to the inference that the erosion resistance of the slope was significantly enhanced by the polymer adhesive material as compared with the control group CK. Moreover, the unimproved SPC of group D-3 and the improved SPC of group E-2 remained intact and firm after scouring by the simulated heavy rain; few particles were eroded and washed away, resulting in a state of almost no soil erosion or loss. According to the results of the erosion resistance test, we can conclude that polymer adhesive is an excellent soil consolidation material, and the addition of polymer composite material can reduce the cement content of SPC without compromising its erosion resistance performance.

#### 3.2.2. Characteristics of Improved SPC

**Figure 9:**
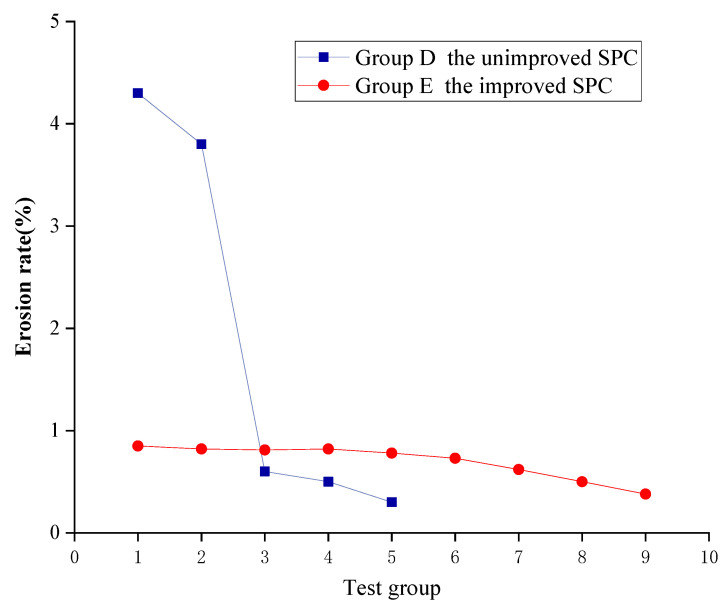
Erosion rates when using SPC before and after improvement.

The erosion resistance rates when using the SPC improved by the polymer composite material reached a level of 0.38–0.85%. As shown in Figure 9, comparing the improved SPC in group E with the unimproved SPC in group D, we found that with the improvement of the polymer composite material the erosion resistance of the SPC in group E reached the same or an even higher level as that of the SPC in group D after the cement content was reduced by about 50%. 

From the perspective of ecological restoration, polymer composite material is greener and more environmentally friendly than cement and can be degraded into CO_2_ and H_2_O. The cement content is the key factor that causes the problems of high alkalinity, overheating, and excessive hardening of SPC, which are not conducive to plant growth. By adding polymer composite material, the cement content of SPC could be reduced by about 50% on the premise of maintaining its excellent erosion resistance performance, which would undoubtedly make it more conducive to plant growth, improve its ecological performance, and allow it to be better applied in the ecological restoration of steep rocky slopes.

### 3.3. Discussion

#### 3.3.1. Improvement Mechanism of Ecological Performance of SPC by Polymer Composite Material

**Figure 10:**
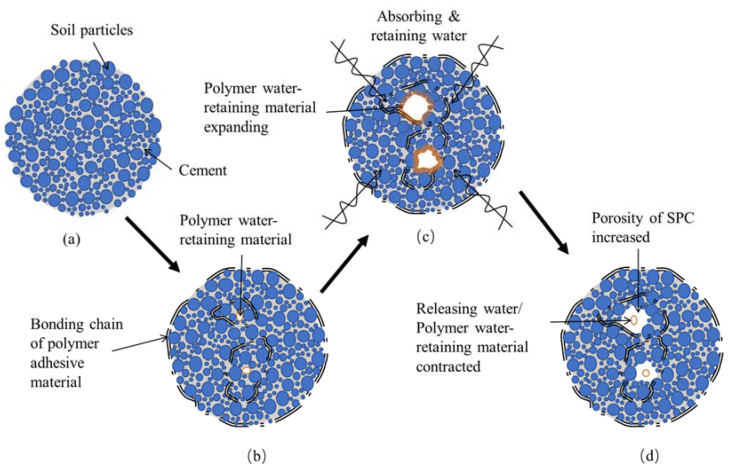
Illustration of improvement mechanism of polymer composite material on SPC: (**a**) the unimproved SPC; (**b**) the molecular chain of the polymer adhesive material improved the stability of SPC; (**c**) water-retaining material expands and exerts extrusion pressure on the surrounding soil; (**d**) the volume expansion and contraction of the polymer water-retaining material leads to an increase in the SPC pores.

The addition of cement makes the internal soil particles of SPC closely consolidated and firm as a whole (as shown in Figure 10a), meaning SPC has superb erosion resistance. However, the addition of cement can inhibit plant growth in SPC and can cause problems of high alkalinity, overheating, and excessive hardening. Therefore, polymer composite material can be used to reduce the cement content in SPC, thereby alleviating these problems and improving SPC’s pore structure and porosity, preventing it from excessive hardening, improving the plant growth environment, and enhancing its ecological performance.

The molecular chain of the polymer adhesive material contains a large number of hydrophilic groups (-OOCCH_3_), which can bind with the cations in the soil and establish physical and chemical bonds between the soil particles to connect the soil particles and make them form soil aggregates [34] (as shown in Figure 10b), which can improve the stability and erosion resistance performance of the soil [35].

It can be seen from the results of the erosion resistance test that the erosion resistance of the soil samples was significantly enhanced under the improvement of the polymer adhesive material [31,35], and the addition of polymer adhesive materials did not produce any substances harmful to the soil environment. Hence, in terms of ecological slope protection, polymer adhesive material could well replace the role of cement in SPC, which can reduce the cement content of SPC and improve its ecological performance while maintaining its excellent erosion resistance performance.

**Figure 11:**
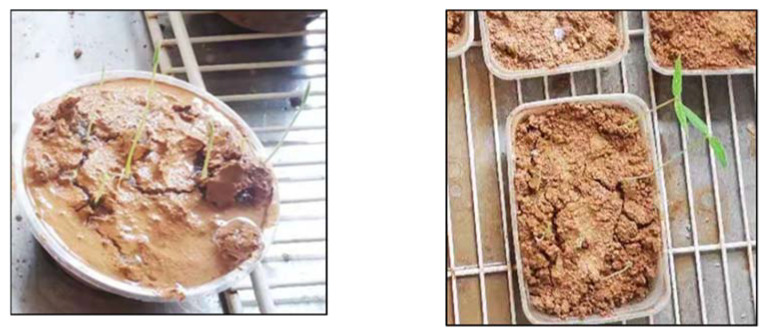
The expansion of water-retaining material increased the bulkiness of soil.

The polymer water-retaining material can store a large amount of water in its molecular long chain and absorb and retain water when the water in the environment is sufficient. During this process, the water-retaining material expands greatly and exerts extrusion pressure on the surrounding soil (as shown in Figure 10c). When the soil moisture gradually decreases, the water in the polymer water-retaining material is released slowly under the adsorption force of the surrounding soil particles and contracts near its original volume [26]. The volume expansion and contraction of the polymer water-retaining material during the water absorption and release process lead to an increase in the SPC pores (as shown in Figure 10d), thus playing the role of fluffing the SPC; improving the soil’s porosity and the permeability of the SPC (as shown in Figure 11) and avoiding the problem of the excessive hardening of the SPC. Moreover, the addition of the water-retaining material enhances the SPC’s water retention and storage capacity, creating a better condition for plant growth and development. In summary, SPC improved by the addition of polymer composite material can satisfy the requirements of erosion resistance and water and soil loss, and this can improve its ecological performance to a higher level so that the SPC can become a good ecological restoration material for steep rocky slopes.

#### 3.3.2. Improvement Effects of Polymer Composite Material on SPC

To further explore the improvements of polymer composite material on SPC, a range analysis, correlation analysis, and multiple regression analysis were carried out using the test results of the unimproved SPC in group D and the improved SPC in group E. The multiple regression equation was established to study the improvement laws of polymer composite material on SPC, which can play an important guiding role in the practical application and promotion of improved SPC.

In the range analysis, indicator $\bar{K}$ reflects the influence of the test material on the test index such as erosion resistance performance, which can determine the optimal dosage of the test material and the optimal material ratio. As for the indicator R, the larger the R-value, the greater the influence of the test material on the test index and therefore, the more influential it is. The indicator R can be used to determine the influence of the test material on the test index from large to small.

The range analysis was carried out on the erosion resistance test results of groups D and E. The influence of the added polymer adhesive material, polymer water-retaining material, and cement on the SPC’s erosion resistance performance was explored and the optimal ratio of these materials was obtained. The results are shown in Table 5.

From the range analysis results, the influence of the three materials on the erosion resistance performance of SPC was as follows, from large to small: cement > polymer adhesive material > polymer water-retaining material. To improve the erosion resistance performance of SPC, the optimal material ratio was found to be the material ratio of test group E-9. Compared with cement, according to the analysis, the polymer adhesive material also showed a strong soil consolidation capacity and can play a significant role in enhancing the erosion resistance performance of SPC. 

A range analysis of the results of the plant growth test for groups D and E was carried out to analyze the impact of the addition of polymer adhesive material, polymer water-retaining material, and cement on the plant growth environment of the SPC, and the optimal ratio of material for use in SPC was obtained. The range analysis results are shown in Table 6. 

As the range analysis results show, the influence of the three materials on the improvement of the plant growth of SPC was as follows, from large to small: polymer water-retaining material > polymer adhesive material > cement. For plant growth, the optimal material ratio was that of test group E-3. From the analysis results, it can be inferred that the polymer adhesive material and the water-retaining material are more conducive to plant growth and development than the cement.

The regression equation was established at a 95% confidence interval to further explore the effects of polymer composite material on the improvement of SPC, taking the additional amounts of polymer adhesive material, cement, and polymer water-retaining material as variables x_1_, x_2,_ and x_3_, and the erosion rate, germination rate, and plant height when using SPC as dependent variables y_1_, y_2_, and y_3_, y = Ax_1_ + Bx_2_ + Cx_3_ + D, A, B, and C are the correlation coefficient. The analysis result is shown in Table 7. The relationship between the dependent variable x and the independent variable y of all the regression equations reached a very significant level (*p*-value < 0.01 or *p*-value < 0.05). The dependent variable x had a significant impact on the independent variable y, meaning all the regression equations were valid.

It can be inferred from the regression equation that the polymer adhesive material x_1_ plays a role in improving the plant growth conditions and erosion resistance performance of SPC; the cement x_2_ is conducive to the erosion resistance performance of SPC but unfavorable to plant growth and the polymer water-retaining material x_3_ is disadvantageous to the erosion resistance performance of SPC but can significantly improve the plant growth of SPC. In particular, it can be seen from Formula (3) that the soil consolidation effect of the polymer adhesive material x_1_ per unit content is even better than that of the cement x_2_ per unit content. It can be deduced from Formula (4) and (5) that the polymer composite material can greatly improve the plant growth environment of SPC. In conclusion, the polymer composite material can improve SPC’s ecological performance and allow it to become an excellent material for slope ecological restoration with superb erosion resistance that is favorable to plant growth.

**Figure 12:**
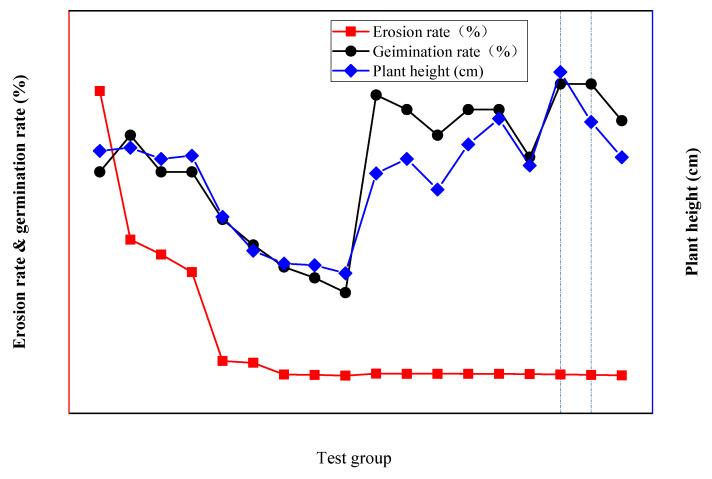
Improvement of SPC with the addition of polymer composite material.

Summarizing the above experimental study and discussion, evidently, there is an optimal material ratio for the improvement of SPC with the addition of polymer composite material (as shown in Figure 12). The erosion rate of the improved SPC in test groups E-7 and E-8 was low, and the germination rates and plant heights were high, which means that the improved SPC in test groups E-7 and E-8 demonstrated outstanding erosion resistance capacity and good ecological performance. While the erosion resistance performance of the improved SPC in group E-6 was also superior, its ecological performance was subpar. When using polymer composite materials to improve SPC, it is necessary to comprehensively consider the relevant situation and select an optimal material ratio according to the relevant test results. Considering the requirements for erosion resistance capacity and plant growth in this study, the optimal material ratio of the improved SPC in South China was found to be that of test group E-7, which is 7% cement, 0.5% polymer adhesive material, and 0.25% polymer water-retaining material in the outside soil.

## 4. Conclusions

1. This study used polymer composite material to improve SPC. A simulated erosion resistance test and plant growth test were carried out to explore the effects, laws, and mechanisms of improvement. The ecological performance of SPC was significantly improved while maintaining excellent erosion resistance performance.
2. Improved by the addition of polymer composite material, the cement content of SPC could be reduced by about 50%. The improved SPC demonstrated superb erosion resistance; the plant germination rate was increased by 200–300%; and the plant height was also significantly improved. SPC improved by the addition of polymer composite material makes a good ecological restoration material for steep rocky slopes.
3. We studied the improvement effects of adding polymer composite material to SPC, which can provide valuable theoretical data and serve as a reference for the application and promotion of SPC in the ecological restoration of steep rocky slopes.

## Figures and Tables

**Table 1:** Physical properties of the tested soil sample.

Saturated Moisture Content (%)	Dry Density (g/cm^3^)	Limit of Liquidity (%)	Limit of Plasticity (%)	Plastic Index of Clay
42.3	1.93	23.7	16.4	15

**Table 2:** Addition of material to the control group and test groups A, B, and C.

Group	Polymer Adhesive Material (%)	Cement (%)	Polymer Water-Retaining Material (%)
CK	0	0	0
A-1	0.5	0	0
A-2	0.75	0	0
A-3	1	0	0
B-1	0	0	0.15
B-2	0	0	0.25
B-3	0	0	0.35
B-4	0	0	0.5
B-5	0	0	0.75
B-6	0	0	1
B-7	0	0	2
C-1	0.5	0	0.25
C-2	0.75	0	0.25
C-3	1	0	0.25
C-4	0.5	0	0.35
C-5	0.75	0	0.35
C-6	1	0	0.35
C-7	0.5	0	0.5
C-8	0.75	0	0.5
C-9	1	0	0.5

**Table 3:** Addition of material to SPC.

Group	Polymer Adhesive Material (%)	Cement (%)	Polymer Water-Retaining Material (%)
D-1	0	3.3	0
D-2	0	4	0
D-3	0	7	0
D-4	0	8.3	0
D-5	0	10	0
E-1	0.5	3.3	0.25
E-2	0.75	3.3	0.35
E-3	1	3.3	0.5
E-4	0.5	4	0.25
E-5	0.75	4	0.35
E-6	1	4	0.5
E-7	0.5	7	0.25
E-8	0.75	7	0.35
E-9	1	7	0.5

**Table 4:** Slope classification by inclination.

Inclination (°)	≤15	15–30	30–70	70–90	≥90
Classification	Mild slope	Moderate slope	Steep slope	Sharp stiff slope	Adverse slope

**Table 5:** Range analysis of erosion resistance test of improved SPC.

Evaluating Indicator	Polymer Adhesive Material (%)	Cement (%)	Polymer Water-Retaining Material (%)
$\bar{K1}$	0.8	0.8	0.6
$\bar{K2}$	0.7	0.8	0.6
$\bar{K3}$	0.6	0.5	0.5
R	0.2	0.3	0.1

**Table 6:** Range analysis of plant growth test when using improved SPC.

Evaluating Indicator	Polymer Adhesive Material (%)	Cement (%)	Polymer Water-Retaining Material (%)
$\bar{K1}$	14.9	17.6	15.4
$\bar{K2}$	16.8	17.4	17.2
$\bar{K3}$	18.4	15.1	19.4
R	3.5	2.5	4.0

**Table 7:** The correlation coefficient of the regression equation.

y	A	B	C	D
y_1_	−0.49 **	−0.09 **	0.01 **	1.31 **
y_2_	0.3 **	−3.14 **	21.1 **	79.6 **
y_3_	3 **	−0.71 **	10.86 *	15 **

Note. ** indicates that the *p*-value of the variable is lower than 0.01, and * indicates that the *S*-value of the variable is lower than 0.05.

## Data Availability

Not applicable.
